# Comparative Craniodental Morphology of Two Endemic Fossil *Sus* Species (Suidae, Mammalia) From the Middle Pleistocene of Java (Indonesia)

**DOI:** 10.1002/jmor.70057

**Published:** 2025-05-24

**Authors:** Rachel V. Pacheco‐Scarpitta

**Affiliations:** ^1^ Department of Earth Sciences Utrecht Universiteit Utrecht the Netherlands

**Keywords:** dentognathic anatomy, Kedung Brubus, *Sus brachygnathus*, *Sus macrognathus*, Trinil H.K

## Abstract

Over a century ago, Dutch anatomist and geologist Eugène Dubois discovered the famous “Java man” and associated mammals in Java. His collection continues to be widely recognised for its significance to palaeontology and palaeoanthropology. Mammal fossil remains from Dubois’ collections have been essential for understanding faunal migrations driven by Quaternary glacial cycles from Southeast Asia to the Sunda Shelf and beyond, and thus the evolution and present distribution of mammals across Island Southeast Asia (ISEA). An important group are the Suinae (pigs). Most extant Eurasian Suinae species belonging to the genus *Sus*, except the widely distributed *Sus scrofa*, are mostly found in ISEA, and represent an example of species radiation. Knowledge of the origin, migration, and evolution of the genus *Sus* is limited, and studies on ecomorphological disparity and phylogeny of fossil Suinae are scarce. Considering the importance of ISEA in the evolutionary history of the genus, a detailed understanding of the fossil *Sus* species from the region is key to understanding the origin, dispersal, and evolution of *Sus*. Here, I focus on the anatomy of two endemic species from the Middle Pleistocene of Java (Indonesia), *S. brachygnathus* and *S. macrognathus*. A detailed anatomical description and morphological comparison between these species and extant and fossil suids are provided, including hitherto undescribed features of two species in the context of ecomorphology. Finally, aspects of the phylogenetic relationships of both species are discussed in relation to insular evolutionary trends. The importance of these fossil *Sus* remains from Java lies not only in their key role to understanding the evolutionary history and diversification of *Sus*, but also in providing insights into the evolutionary trends of insular pigs.

## Graphical abstract

Two endemic fossil pig species, *Sus brachygnathus* and *S. macrognathus*, from the Middle Pleistocene of Java are described in detail. Hitherto undescribed anatomical features of both species are discussed in the context of ecomorphology and phylogenetic relationships. This study constitutes a firm morphological basis for future phylogenetic and taxonomic studies in both extant and extinct *Sus* based on craniodental morphology.

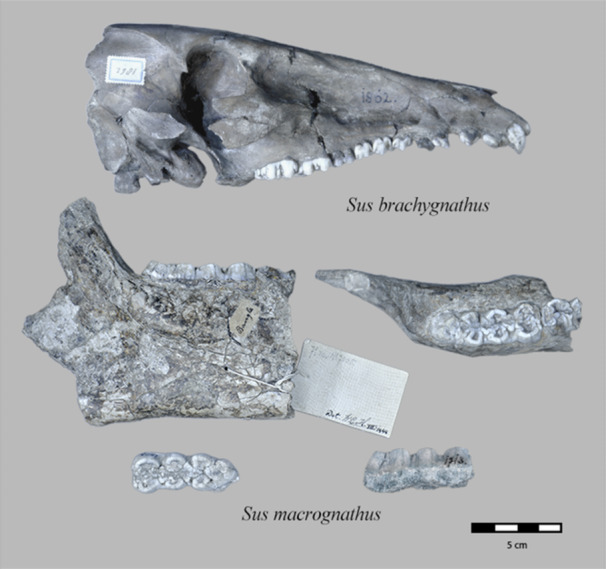

## 1 Introduction

The Suidae (pigs, hogs, and their allies) is a well‐represented family of even‐toed ungulates (Artiodactyla) in the Eurasian Cenozoic fossil record, though they are generally less common than other Artiodactyla taxa. The earliest putative suid remains (*Egatochoerus*, *Eocenchoerus, Siamochoerus* and *Odoichoerus*) date back to the late Eocene in Asia (Tong and Zhao 1986; Ducrocq 1994; Liu and Huang 2001; van der Made 2010; Orliac et al. 2011; Ducrocq et al. 2024). By the middle Miocene ( ~ 15 Ma), Suidae had diversified considerably, dispersing into the rest of Eurasia and Africa (Fortelius et al. 1996; Pickford 2012). The subfamily Suinae emerged during the late Miocene and became dominant after, while other subfamilies went extinct in Eurasia ( ~ 10 Ma) within the context of the Vallesian Crisis (Iannucci and Begun 2022; van der Made et al. 2022). By the beginning of the Pliocene, all subfamilies of Suidae other than Suinae and Tetraconodontinae had already gone extinct (Frantz et al. 2016). Suinae includes all extant suids, except for the enigmatic species *Babyrousa babyrussa* Linnaeus 1758, which is sometimes placed in a subfamily on its own (Babyrousinae). In Asia, Suinae is represented by the genera *Sus* and *Porcula*, of which the latter is monospecific with a limited distribution (Frantz et al. 2016; Melletti and Meijaard 2017).

Little is known about the origin, dispersal and evolution of *Sus* species. The earliest known species of *Sus* is *Sus arvernensis* Croizet and Jobert 1828, which is dated to the Pliocene of continental Eurasia (van der Made et al. 2006; Cherin et al. 2018; Iannucci and Begun 2022; Iannucci et al. 2022; Iannucci 2024). This species is considered ancestral or closely related to the ancestor of numerous extinct and living lineages, including the Early Pleistocene *Sus strozii* Forsyth Major 1881 from Europe, the Sardinian dwarf *Sus sondaari* van der Made 1999 and the living warty pigs of ISEA (Iannucci 2024). Nevertheless, some authors, drawing mostly from molecular data, have proposed an East Asian origin of the genus (Frantz et al. 2013). Based on mtDNA of modern Eurasian *Sus*, Larson et al. (2005) reconstructed a phylogeographical framework that indicated that the genus originated in ISEA, from where it dispersed across continental Eurasia.

Research on the phylogenetic relationships between extant and fossil Suinae is scarce and restricted to a few species of *Sus* (Liu 2003; Pickford 2012), except for Hardjasasmita (1987) and Cherin et al. (2018), who included a total of eight and nine species of *Sus*, respectively. Consequently, the phylogeny of the genus is still debated, which is reflected in the taxonomy of the living species. For living taxa, some authors (Groves 1981; van der Made 1996; Pickford 2012) divide the genus in two main groups: “scrofic” and “verrucosic” type based on the morphology of the cross‐section of the lower canine in males. The scrofic group includes *Sus scrofa* Linnaeus 1758 whilst the verrucosic group includes all other living species, including *S. verrucosus* Boie 1832, *S. barbatus* Müller 1838 and *S. celebensis* Müller and Schlegel 1843, among others. However, the phylogenetic separation based on this single trait has been doubted (Groves 1997; Lucchini et al. 2005). Phylogeny based on molecular data situates *S. scrofa* as the basal taxon of *Sus*, followed by the radiation of ISEA suids (Frantz et al. 2016; Gongora et al. 2011). Fossil evidence of *Sus* species from continental Eurasia also includes the previously mentioned European *S. strozii* (Cherin et al. 2018), *S. xiaozhu* Han et al. 1975 (Hu et al. 2023; Dong et al. 2013) and *S. lydekkeri* Zdansky 1928 (Liu et al. 2017) from North China, among others. However, all extant wild *Sus* other than *S. scrofa* are limited to Island South‐East Asia (ISEA), including *S. barbatus* (Borneo, Sumatra and Malay Peninsula), *S. verrucosus* (Java) and *S. celebensis* (Sulawesi), among others. Since the late Pliocene, *Sus* has diversified into at least seven morphologically defined species in ISEA (Frantz et al. 2016, 2013; Pickford 2012), suggesting independent allopatric speciation events. This rapid radiation was probably initiated by the dispersal of *Sus* from the Asian mainland to the Sunda Shelf islands, followed by the subsequent isolation of these populations. These events of dispersal‐isolation were probably driven by Pleistocene large‐scale climatic fluctuations, characterized by successive glacial and interglacial periods that caused dramatic changes in sea level, thus alternately isolating and connecting large landmasses along the Sunda Shelf (van den Bergh et al. 1996; van den Bergh et al. 2001; van der Geer et al. 2021). Also, these changing climate conditions require rapid adaptations that can lead to parapatric speciation. The region of ISEA thus constitutes a key area for understanding the origin, dispersal, and diversification of *Sus* since the Pleistocene. Furthermore, morphological insights are important for resolving the relationships between ISEA pigs, given the recurrent admixture events during the Pleistocene, driven by the sea‐level oscillations, which resulted in some contrasting molecular data. However, the phylogeny and evolutionary ecology of *Sus* remain insufficiently known. An essential step to gaining new insights into the evolution of *Sus* is to provide a detailed record of the fossil *Sus* species from ISEA considering their ecomorphological adaptations in comparison with extant and fossil suid species.

Fossil remains of the Dubois collection from Java (Indonesia) are particularly interesting in this respect because of their pivotal role in understanding the Pleistocene main faunal migration events from mainland Southeast Asia to the Sunda shelf and consequently, their subsequent evolution and current biogeography (Aziz et al. 1995). In Java, two Pleistocene *Sus* species are known. They were first described by Dubois (1908): *Sus brachygnathus* from the older site Trinil H.K. and *S. macrognathus* from the younger site Kedung Brubus, both dating to the Middle Pleistocene but not contemporaneous. Badoux (1959), based on comparison and morphological analysis of the teeth of these species, rejected the validity of Dubois’ species, arguing that S*. brachygnathus* is equivalent to extant *S. barbatus* and *S. macrognathus* to extant *S. verrucosus*. Hardjasasmita (1987), however, based on skull and tooth macroscopical traits, considered Dubois’ species valid, assigning a lectotype to each species and providing a concise description of the material. In the present work, I provide new and comprehensive anatomical descriptions of these two Javanese fossil species along with an extensive morphological comparison between these and extant Suinae species worldwide. I here compare these two species with fossil *Sus* species from the Pleistocene of China and with the European *S. strozii* from the Pleistocene of Europe. Finally, phylogenetic aspects are discussed in the context of the phylogenies of Cherin et al. (2018) and Cucchi et al. (2009). The main aim is to contribute to a firm morphological basis for future phylogenetic and taxonomic studies of *Sus*.

## 2 Material and Methods

All fossil material of *S. brachygnathus* and *S. macrognathus* described here is part of the Dubois’ collection (Naturalis Biodiversity Center, Leiden, the Netherlands) (Table 1) from Trinil H.K. and Kedung Brubus biozones (van den Bergh et al. 2001; van der Geer et al. 2021; Huffman et al. 2022), respectively.

**Table 1:** List of the fossil material described (rows 1–2), the comparative specimens of extant suids considered for this study (rows 3–12) and extinct *Sus* (rows 13–16).

Scientific name	Common name	Catalogue number	Type of material	Locality/Origin	Museum
*Sus brachygnathus*	—	RGM. DUB.1847	Complete female mandible	Trinil (Java, Indonesia)	Naturalis
		RGM. DUB.1848	male hemimandible sin. C‐M_3_		
		RGM. DUB.1854	Male hemimandible dex I^3^‐M^3^		
		RGM. DUB.1860	Male skull		
		RGM. DUB.1862	Female skull		
*Sus macrognathus*	—	RGM. DUB.39a	Maxilla sin. P^3^‐P^4^	Kedung Brubus (Java, Indonesia)	
		RGM. DUB.39b	Maxilla dex. P^4^‐M^1^		
		RGM. DUB.39c	Mandible dex P_3_‐P_4_		
		RGM. DUB.39e	M^3^ sup dex.		
		RGM. DUB.7005a	Mandible sin. M_3_		
		RGM. DUB.1713 lectotype	M_3_ inf sin.		
*Sus barbatus*	Bornean bearded pig	RMNH. MAM.39259	Complete skull	Indonesia	
*Sus celebensis*	Celebes warty pig	ZMA. MAM.1166	Complete female skull		
*Sus celebensis*		RMNH. NAM.n.n.	Complete skull	Simeulue (Indonesia)	
*Sus celebensis timoriensis*		RMNH. MAM. 39256.a	Partial male skull	Indonesia	
*Sus scrofa vitattus*	Banded pig	ZMA. MAM.1157	Complete male skull	Maumere (Indonesia)	
*Sus verrucosus*	Javan warty pig	RMNH. MAM.35336	Complete male skull	Zamanap (Indonesia)	
*Babyrousa babyrussa*	North Sulawesi babirusa	ZMA. MAM.9115	Complete female skull	Indonesia	
*Potamochoerus porcus*	Red River Hog	ZMA. MAM.7382	Complete male skull	Belinga, (Gabon)	
*Potamochoerus larvatus*	Bushpig	RMNH. MAM.1687.a	Complete female skull	—	
*Phacochoerus aethiopicus*	Dessert Warthog	ZMA. MAM.17883	Complete male skull	Kenya	
*Sus lydekkeri*	—	HY13‐58.1HY13.58.2	Complete female skull Complete female mandible	Yangshuizhan (Yueyang, Hunan, southern China)	NNMO
*Sus peii*	—	V18402.9	M^2^ dex.	Sanhe Cave (Chongzuo, Guangxi, North China)	IVPP CAS
		V18402.11	M^3^ sin.		
		V18402.13	M^3^ dex.		
		V18402.17	M_1_ dex.		
		V18402.15	M_1_ sin.		
		V18402.19	M_2_ dex.		
*Sus strozii*	—	SBAU 337647	Male hemimandible sin.	Pantalla (Italy)	SBAU
*Sus xiaozhu*	—	CYM0002 CYM0003 CYM0004 CYM0005	Complete maxilla, partial frontal bone. Partial dex. skull P^4^‐M^2^ Partial mandible Partial hemimandible sin.	Sifangdi (Chongqing, southwest China)	CQKL‐PPC

*Note:* dex, dexter; sin, sinister.

Institutional abbreviations: CQKL‐PPC, Chongqing Key Laboratory of Paleontology and Paleoenvironmental Co‐evolution, China; IVPP CAS, Institute of Vertebrate Paleontology and Palaeoanthropology, Chinese Academy of Sciences; Naturalis, Naturalis Biodiversity Center, Leiden, The Netherlands; NNMO, Nihewan National Nature Reserve Management Office, Hebei, China; SBAU, Soprintendenza per i Beni Archeologici dell'Umbria, Perugia, Italy.

References: *Sus lydekkeri* is in Liu et al. (2017); *Sus peii* in Dong et al. (2013); *Sus strozii* in Cherin et al. (2018) and *Sus xiaozhu* in Hu et al. (2023).

**Table 2:** List of the craniomandibular anatomical terms used in the fossil descriptions along with their English equivalents.

	Anatomical term (Latin)	English equivalents
**Skull**	*Bulla tympanica*	Tympanic bulla
	*Condylus occipitalis*	Occipital condyle
	*Foramen lacrimale*	Lacrimal foramen
	*Foramen magnum*	Foramen magnum
	*Foramen palatinum majus*	Greater palatine foramen
	F*oramen supra/infra‐orbitale*	Supra/infra‐orbital foramen/Infraorbital fossa
	*Fossa temporalis*	Temporal fossa
	*Maxilla*	Maxillary bone
	*Meatus acusticus externus*	External auditory meatus
	*Os nasale*	Nasal bone
	*Os palatinun*	Palatine bone
	*Os zygomaticum*	Zygomatic bone
	*Pars basilaris ossis occipitalis*	Basioccipital bone
	*Protuberantia occipitalis externa*	External occipital protuberance
	*Processus jugularis ossis occipitalis*	Jugular process
	*Processus zygomaticus ossis frontalis*	Zygomatic process of the frontal bone/Zygomatic arch
	*Saccus lacrimalis*	Lacrimal sac
	*Sulcus supraorbitalis*	Supraorbital sulcus
	*Tuberculum articulare ossis temporalis*	Articular tubercle of the temporal bone
	*Tuber maxillae*	Maxillary tuberosity
**Lower jaw**	*Caput mandibulae*	Mandibular head
	*Corpus mandibulae*	Mandibular body
	*Facies buccalis*	Buccal face
	*Facies lingualis*	Lingual face
	*Foramen mandibulae*	Mandibular foramen
	*Foramen mentale*	Mental foramen
	*Fossa masseterica*	Masseteric fossa
	*Fovea pterygoidea*	Pterygoid fovea
	*Incisura mandibulae*	Mandibular notch
	*Linea mylohyoidea*	Mylohyoid line
**Muscle**	*M. masseter*	Masseteric muscle
	*M. mylohyoideus*	Mylohyoid muscle
	*M. pterygoideus lateralis*	Lateral pterygoid muscle
	*M. pterygoideus medialis*	Medial pterygoid muscle
**Lower jaw**	*Margo alveolaris*	Alveolar border
	*Margo interalveolaris*	Alveolar margin
	*Margo ventralis*	Ventral border
	*Pars incisiva*	Incisive part
	*Pars molaris*	Molar part
	*Processus condylaris*	Mandibular condyle
	*Processus coronoideus*	Coronoid process
	*Ramus mandibulae*	Mandibular ramus
	*Symphysis mandibulae*	Mandibular symphysis

*Note:* The colours correspond to the specific anatomical structure related to the terms (indicated also in the first column). Only the English equivalents are used throughout the manuscript.

Pleistocene‐Holocene deposits are commonly found in the Sangiran area (Central Java) and Kendeng Hills (East Java). The fossil localities of Trinil and Kedung Brubus are located along the southern edge of the fold belt of the Kendeng Hills. The remains of *S. brachygnathus* were found at the fossil‐rich beds of Trinil fossil site, known as the “LP/HK unit” because Dubois named it “Lapilli bed” (LP) while Selenka's team (1907) referred to it as the Hauptknochenschicht (HK). Dubois interpreted the LP as alluvial deposits, where the skeletal remains were deposited in an ancient valley by the Solo paleo‐river (Dubois 1894; Berghuis et al. 2021; Huffman et al. 2022). *Sus macrognathus* material is found from the tuffaceous sandstone layer at Kedung Brubus fossil site with hominin (Zaim 2010). Tuffaceous sandstone layer corresponds to the boundary between the Kabuh and underlies the Pucangan Formations (Fm.). The Kabuh Fm. is formed by light grey volcanic clays with coarse and cross‐bedded tuffaceous sandstones and conglomerates, featuring cross‐cutting channel structures that were deposited by braided rivers (Zaim 2010; Huffman et al. 2022).

The material included here consists of mandibles and dental remains (isolated as well as articulated) and two crania of *S. brachygnathus* from a male and a female individual. The measurements of the skull and dental material were taken with vernier calipers, accurate to 0.1 mm. The craniodental measurements follow Janis (1990), and those for *S. brachygnathus* are compiled in Table 3 (molars) and Table 4 (skull and lower jaw), while those for *S. macrognathus* are provided in Table 5 (molars).

**Table 3:** Molar measurements (in mm) of *Sus brachygnathus*.

Specimen number	M_1_L (*N* = 15)	M_1_W (*N* = 15)	M_2_L (*N* = 19)	M_2_W (*N* = 19)	M_3_L (*N* = 14)	M^2^l (*N* = 13)	M^2^W (*N* = 12)
RGM. DUB.41b	—	—	—	—	—	20.0	16.0
RGM. DUB.41 d	15.0	12.0	22.0	14.0	—	—	—
RGM. DUB. 79a	13.1	10.0	19.0	14.1	—	—	—
RGM. DUB.83 g	—	—	—	—	—	19.1	15.1
RGM. DUB.499a	11.5	7.5	15.3	10.8	—	—	—
RGM. DUB.504b	—	—	16.0	14.0	—	—	—
RGM. DUB.511a	—	—	—	—	—	19.2	14.1
RGM. DUB.511b	—	—	—	—	—	22.0	15.0
RGM. DUB.1473b	—	—	17.0	11.3	—	—	—
RGM. DUB.1688a	—	—	16.1	11.0	31.0	—	—
RGM. DUB.1827	12.1	9.0	16.2	12.0	29.0	—	—
RGM. DUB.1834	13.1	8.5	16.1	11.5	28.0	—	—
RGM. DUB.1837a	—	—	—	—	—	19.0	14.0
RGM. DUB.1837b	—	—	—	—	—	20.0	14.0
RGM. DUB.1837c	—	—	—	—	—	18.5	12.0
RGM. DUB.1837d	—	—	—	—	—	19.0	14.2
RGM. DUB.1837f	—	—	—	—	—	19.1	14.1
RGM. DUB.1837h	12.2	9.0	18.0	13.0	—	—	—
RGM. DUB.1837i	12.2	8.2	15.3	12.0	29.0	—	—
RGM. DUB.1837k	—	—	18.0	11.0	—	—	—
RGM. DUB.1837m	—	—	—	—	30.0	—	—
RGM. DUB.1843	12.0	9.1	18.1	12.1	—	—	—
RGM. DUB.1844	12.0	9.1	19.1	12.0	—	—	—
RGM. DUB.1845	10.0	7.1	12.0	10.0	32.0	12.0	—
RGM. DUB.1847	11.0	8.0	14.5	11.5	27.0	—	—
RGM. DUB.1848	12.0	9.0	18.0	12.0	32.0	—	—
RGM. DUB.1855	12.3	8.5	15.0	12.1	32.0	—	—
RGM. DUB.1854	13.0	9.0	19.0	13.0	35.0	—	—
RGM. DUB.1856a	—	—	—	—	25.6	—	—
RGM. DUB.1860	—	—	—	—	—	17.5	15.0
RGM. DUB.1862	—	—	—	—	—	18.0	14.0
RGM. DUB.538a	—	—	—	—	36.0	—	—
RGM. DUB.499b	—	—	—	—	—	17.1	13.3
RGM. DUB.499c	11.1	9.1	16.0	12.0	—	—	—
RGM. DUB.1833	—	—	—	—	31.0	—	—
RGM. DUB.503a	—	—	—	—	37.0	—	—

*Note:* RGM. DUB.1848, RGM. DUB.1854 and RGM. DUB.1862 are paralectotypes. Measurements taken are M_1_L (first lower molar length), M_1_W (first lower molar width), M_2_L (second lower molar length), M_2_W (second lower molar width), M_3_L (third lower molar length), M^2^L (second upper molar length) and M^2^W (second upper molar width).

**Table 4:** Skull and lower jaw measurements (in mm) of *Sus brachygnathus*.

Specimen number	MFL	OCH	PSL	BCL	TSL	PJL	WMA	TJL
RGM. DUB.1845	—	—	—	—	—	—	—	237
RGM. DUB.1843	—	—	—	—	—	48.0	53.0	18.0
RGM. DUB.1848	—	—	—	—	—	70.0	75.0	250
RGM. DUB.1854	—	—	—	—	—	75.0	76.0	255
RGM. DUB.1862	53.0	550	100	70.0	270	—	—	—
RGM. DUB.1860	—	570	—	—	—	—	—	—

*Note:* Specimen RGM. DUB.1862 is a female skull; RGM. DUB.1860 is a male skull. Measurements taken are MFL (length of masseteric fossa), OCH (occipital height), PSL (posterior length of the skull), BCL (basicranial length), TSL (total skull length), PJL (posterior jaw length), WMA (maximum width of mandibular angle) and TJL (total jaw length).

**Table 5:** Molar measurements (in mm) of *Sus macrognathus*.

Specimen number	M_1_L (*N* = 6)	M_1_W (*N* = 6)	M_2_L (*N* = 14)	M_2_W (*N* = 14)	M_3_L (*N* = 25)	M^2^L (*N* = 7)	M^2^W (*N* = 7)
RGM. DUB .38b	—	—	—	—	—	17.0	18.0
RGM. DUB 38 g	—	—	—	—	44.2	—	—
RGM. DUB 38 h	—	—	—	—	44.0	—	—
RGM. DUB 38 f	—	—	—	—	44.0	—	—
RGM. DUB 78 f	—	—	25.0	16.0	—	—	—
RGM. DUB 78e	14.1	10.5	21.2	12.0	43.1	—	—
RGM. DUB 79 f					43.1		
RGM. DUB 83 d	15.1	12.0	20.0	15.0	43.0	—	—
RGM. DUB 83e	—	—	21.2	15.0	43.0	—	—
RGM. DUB 80	—	—	22.0	14.0	41.3	—	—
RGM. DUB 84a	—	—	—	—	42.5	—	—
RGM. DUB 114a	—	—	—	—	—	24.1	19.0
RGM. DUB 114c	—	—	—	—	41.0	—	—
RGM. DUB 114e					44.0		
RGM. DUB 497a	14.2	9.5	20.1	12.0	42.0	—	—
RGM. DUB 538c	—	—	—	—	—	25.0	15.1
RGM. DUB 714a	—	—	25.0	15.0	—	—	—
RGM. DUB 714 d	—	—	24.0	17.0	—	—	—
RGM. DUB 714e	—	—	—	—	44.0	—	—
RGM. DUB 715a	—	—	—	—	46.0	—	—
RGM. DUB 715b	—	—	—	—	36.4	—	—
RGM. DUB 715c	—	—	—	—	—	23.3	17.0
RGM. DUB 716a	—	—	24.0	15.0	41.0	—	—
RGM. DUB 716b	—	—	—	—	43.0	—	—
RGM. DUB 716 d	—	—	—	—	—	24.0	15.2
RGM. DUB 716 g	—	—	22.0	12.0	—	—	—
RGM. DUB 1713	—	—	—	—	48.0	—	—
RGM. DUB 1835d	—	—	—	—	—	25.0	18.1
RGM. DUB 1835e	—	—	—	—	42.0	—	—
RGM. DUB 1835g	—	—	21.1	15.0	—	—	—
RGM. DUB 1838 a	—	—	21.1	16.0	47.0	—	—
RGM. DUB 1838b	14.0	10.0	21.3	14.0	43.0	—	—
RGM. DUB 1840b	—	—	—	—	42.1	—	—
RGM. DUB 1840c	25.0	16.0	—	—	—	—	—
RGM. DUB 1844	—	—	—	—	—	23.1	17.0
RGM. DUB 6980	—	—	—	—	37.3	—	—
RGM. DUB 7005b	15.0	10.0	23.0	14.0	—	—	—
RGM. DUB 7005c	—	—	—	—	38.0	—	—
RGM. DUB 7005a	—		—	—	47.0	—	—

*Note:* RGM. DUB.1713 is the lectotype. Measurements taken are the same as those of *Sus brachygnathus*, indicated in Table 1.

The anatomical nomenclature used in this study follows Barone (2000, 2010) and Gasse (2017). For the anatomical terms used for the skull descriptions, see Table 2. All Latin terms are provided for clarity, but only the English equivalents are used in the text for simplicity and consistency. The dental terminology used here is based on van der Made (1996) as modified by Cherin et al. (2018).

The two Javanese fossil suid species described here were compared with a total of 12 suid species (8 extant, 4 fossil) (see Table 1 for specimens). The extant species include some of the Island Southeast Asia (ISEA) wild pigs: *S. barbatus*, *S. verrucosus, S. celebensis* and *S. scrofa vittatus;* three species of African suids: *Potamochoerus porcus*, *P. larvatus* and *Phacochoerus aethiopicus* and the enigmatic *Babyrousa babyrussa*. The fossil species selected for the comparison were *S. xiaozhu*, *S. peii* Han 1987, *S. lydekkeri* from the Pleistocene of China and the European *S. strozii*, for which morphological data was taken from published works (see references in Table 1).

## 3 Results

### 3.1 Sus brachygnathus

#### 3.1.1 Systematic Palaeontology

Order Artiodactyla Owen 1848.

Family Suidae Gray 1821.

Subfamily Suinae Gray 1821.

Genus Sus Linnaeus 1758

Species *Sus brachygnatus* Dubois 1908

**Figure 1:**
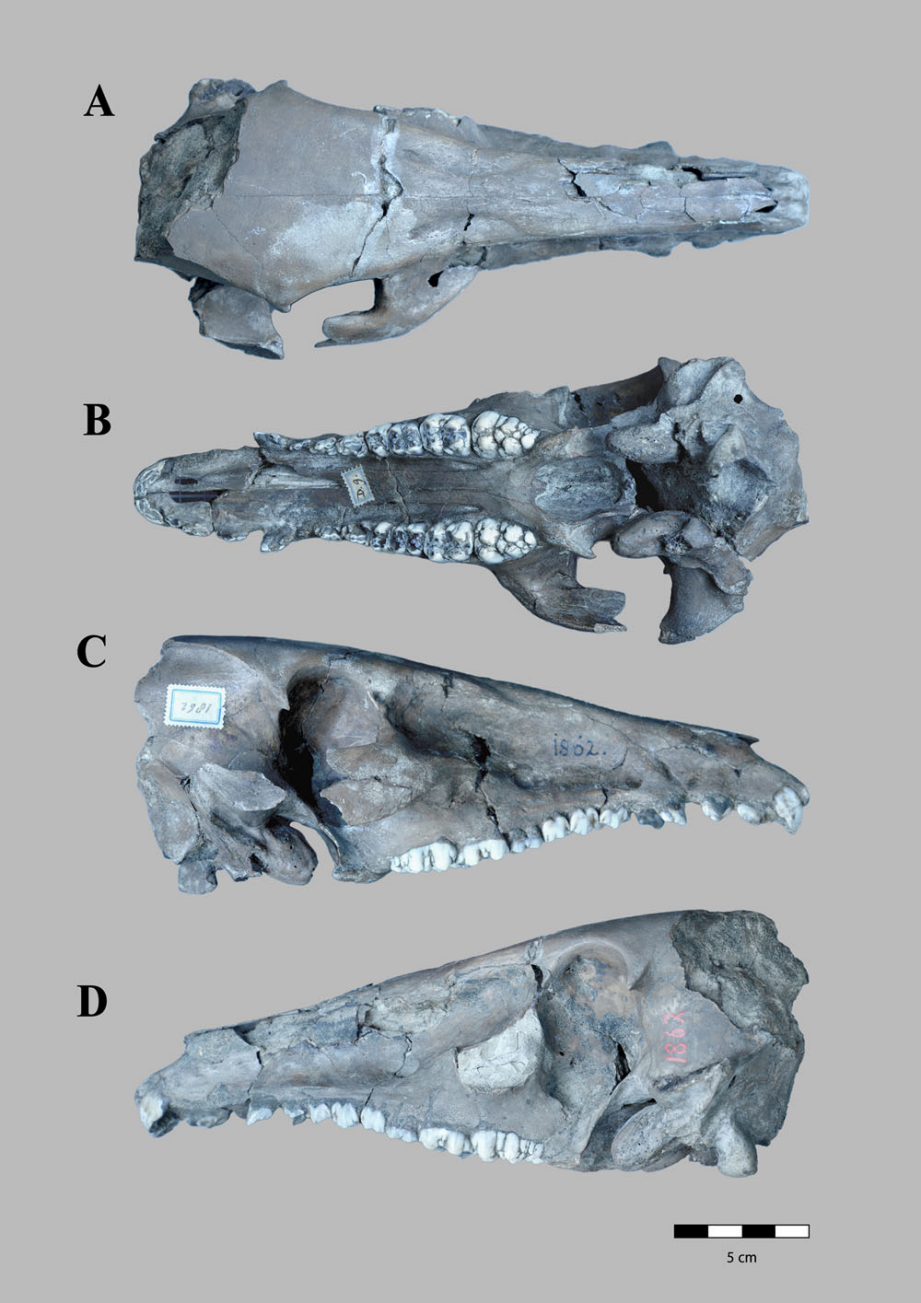
*Sus brachygnathus* female skull (RGM. DUB.1862) from Trinil, Java (Indonesia; Middle Pleistocene). A) dorsal, B) ventral, C) lateral (right side), and D) lateral (left side) views.

**Figure 2:**
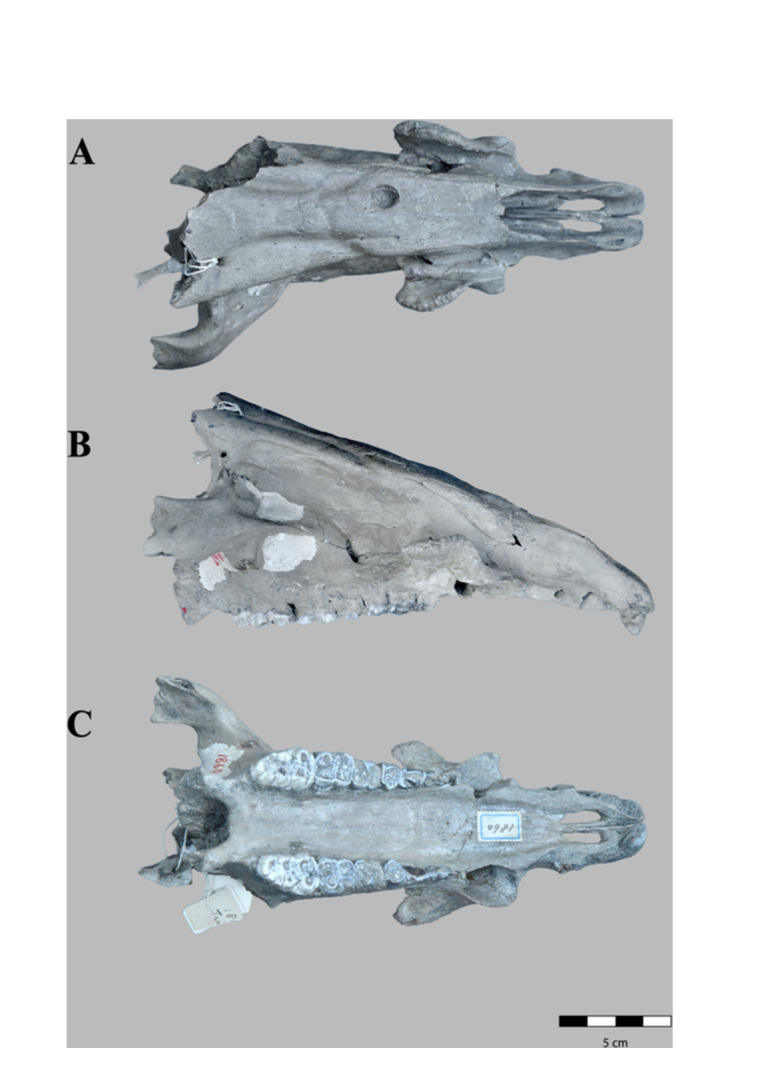
*Sus brachygnathus* male skull (RGM. DUB.1860) from Trinil, Java (Indonesia; Middle Pleistocene). A) dorsal, B) lateral and C) ventral views.

**Figure 3:**
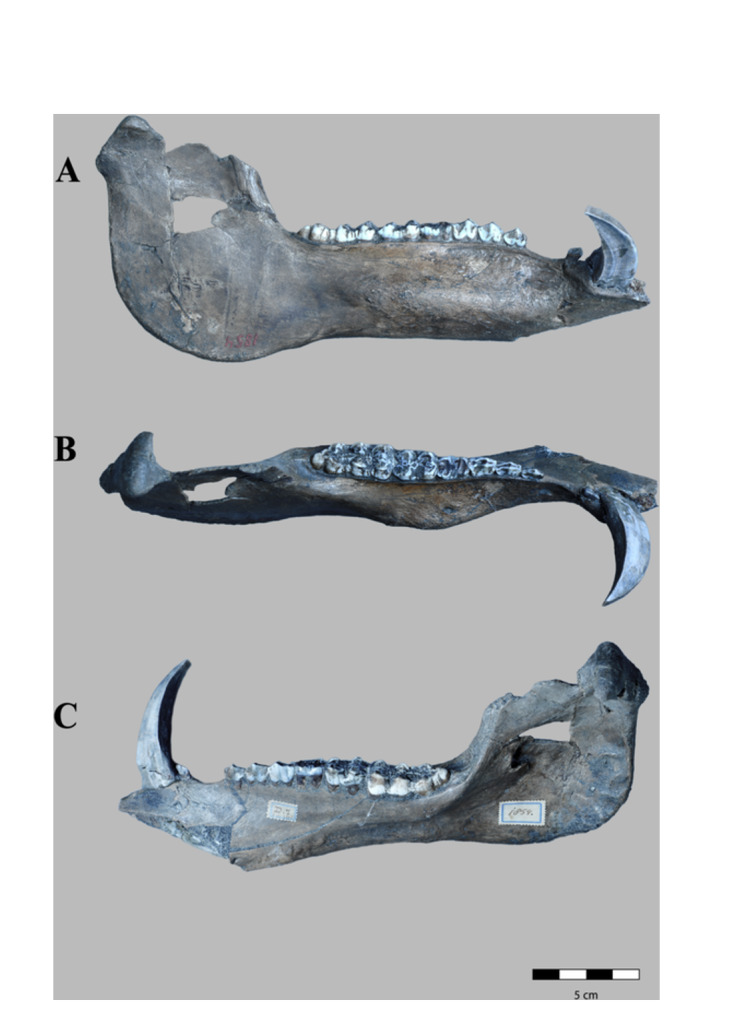
*Sus brachygnathus* right hemimandible of an adult male (RGM. DUB.1854) from Trinil, Java (Indonesia; Middle Pleistocene). A) lateral, B) dorsal and C) medial views.

**Figure 4:**
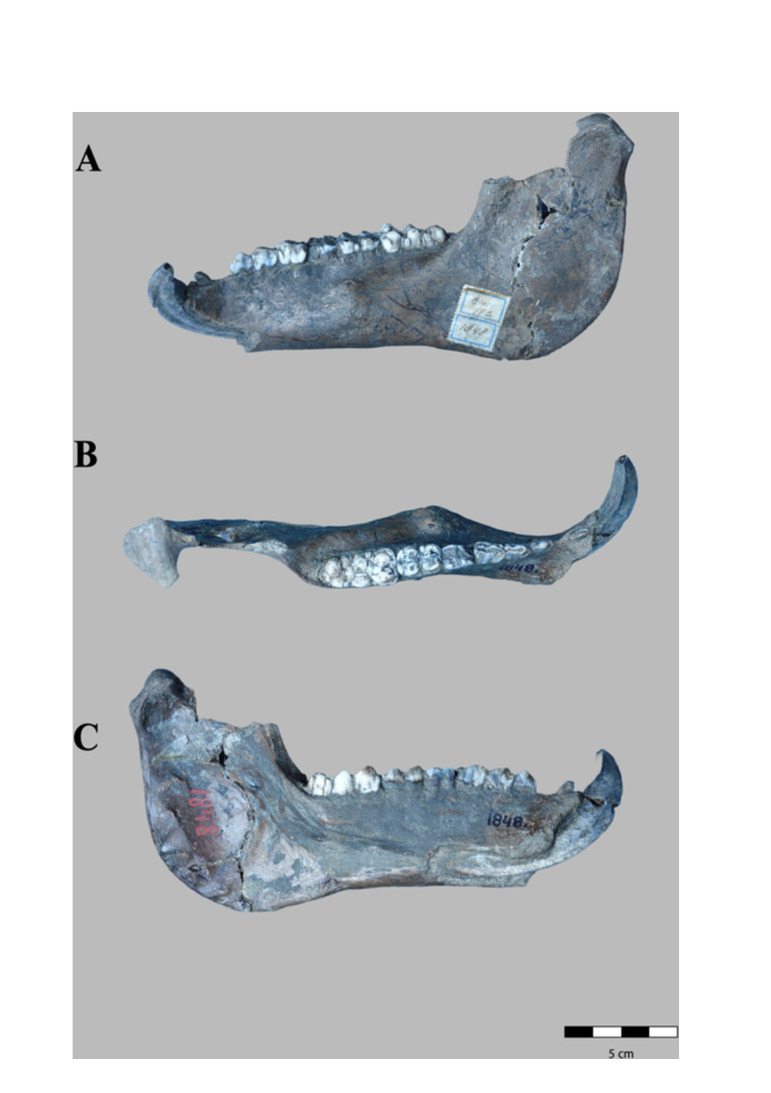
*Sus brachygnathus* left hemimandible of an adult male (RGM. DUB.1848,) from Trinil, Java (etc.). A) lateral, B) dorsal and C) medial views.

**Figure 5:**
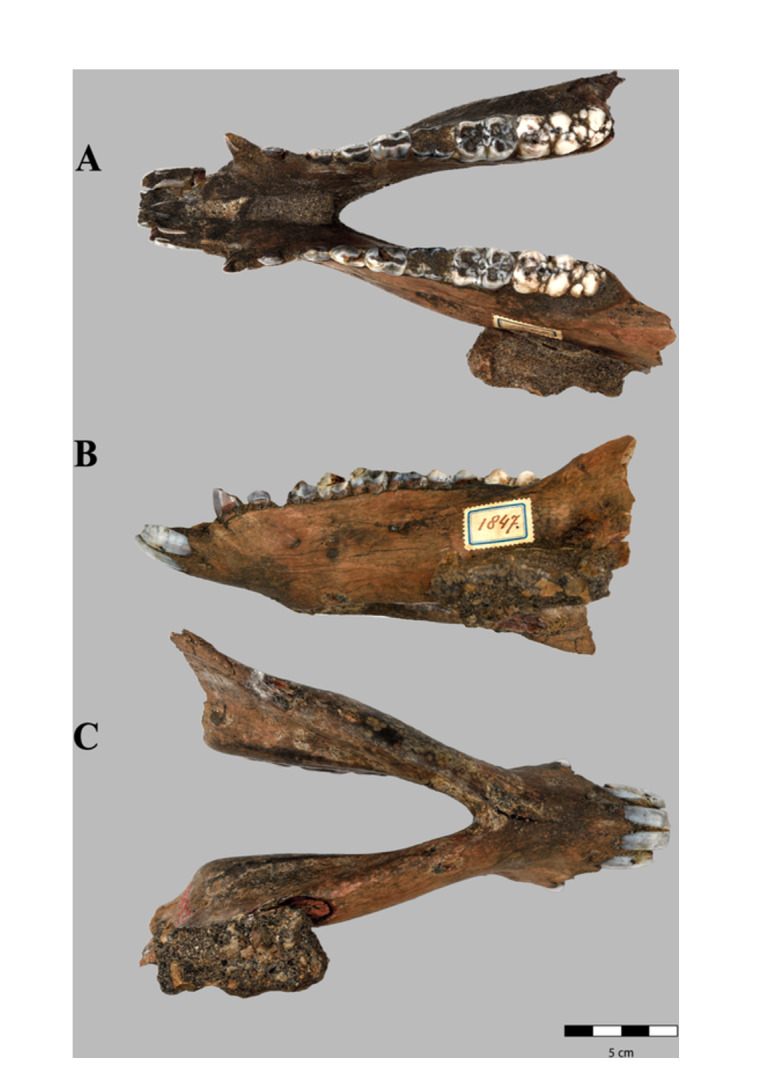
*Sus brachygnathus* complete mandible of an adult female (RGM. DUB.1847) from Trinil, Java (etc.). A) dorsal, B) lateral, and C) ventral views.

Figures 1, 2, 3, 4, 5, Table 3.

Synonyms: S*us hysudricus* Martin 1890.

*Sus celebensis* Dubois 1907.

*Sus brachygnathus* Dubois 1908

*Sus macrognathus* Stremme 1911, part.

*Sus vatualangensis* von Koenigswald 1933.

*Sus barbatus* Badoux 1959.

*Sus brachygnathus* Hardjasasmita 1987.

#### 3.1.2 Known Distribution

Type Locality: Trinil, Java

Age: Late Early Pleistocene/Early Middle Pleistocene (~ 0.9‐ 0.78 Ma).

Occurrence: Trinil, Bangle, Tritik, Pati Ajam, Kedung Doeren, Tandieng, Tegoean, and Kedung Pring.

#### 3.1.3 Material Examined

Lectotype (not examined in this study): RGM. DUB.1861, designated by Hardjasasmita 1987

RGM. DUB.1847 (complete female mandible; no type); 1848 (hemimandible sin c‐M_3_); 1854 (hemimandible dex I_3_‐M_3_); 1862 (complete skull sin I^1^‐M^3^ dex I^1^‐M^3^) and 1860 (male skull; no type). Specimens RGB. DUB.1862, 1854, and 1848 are referred to as syntypes 1‐ 3 in Hardjasasmita (1987), but are considered paralectotypes, following the designation of the RGM. DUB.1861 as the lectotype.

#### 3.1.4 Revised Diagnosis

Small‐sized fossil pig with flattened skull and long straight‐shaped snout. Overall, the skull morphotype is no big difference to the extant species *Sus celebensis*. The parietal‐occipital region is markedly low in comparison with extant suid species considered for this study. The foramen magnum is situated in a ventral position. The lower male canine is of the verrucosic type. Incisors are wide and high‐crowned. Premolars are large, well‐developed, and have a cutting surface. Molars are bunodont and brachydont in terms of morphology, and with a high number of accessory cusps. The talonid of the M_3_ is comparable with the extant pigs *S. barbatus*. Dental formula: I 3/3, C 1/1, PM 4/4 and M 3/3.

### 3.2 Anatomical Descriptions

- RGM. DUB.1862. *Sus brachygnathus*; paralectotype; female skull, complete, sin I^1^‐M^3^ dex I^1^‐M^3^. Figure 1.

This skull belongs to a young adult female. The skull is complete for the larger part and well‐preserved. The right zygomatic arch is preserved except for a part of the central portion, and is missing on the left side. The top of the nuchal crest and almost the entire parietal bone are missing, as well as the basioccipital bone and the external occipital protuberance. Parts of the nasal bone are absent. The right P^2^ and P^3^ are missing.

The skull is relatively elongated, similar to that of *S. celebensis* in size and morphology but considerably shorter than in *S. verrucosus*, *S. barbatus*, *S. scrofa vitattus*, and *Potamochoerus*. It is flattened dorsoventrally as in *S. celebensis*, and with a narrow and long muzzle. Unlike *S. celebensis*, *S. brachygnathus* has a straight profile from the nasal bone to the parietal bone. Considering its flattened profile of the skull in the lateral view and the low position of the parietal bone, the occipital region and nuchal crest do not seem to extend much upwards. *S. brachygnathus* has the lowest upper occipital region of the eight extant suid species analysed here.

#### 3.2.1 Dorsal Face

The skull has a triangular shape. The lateral borders of the nasal bone run parallel. The zygomatic process of the frontal bone can be distinguished as a small prominence that extends ventrally on both sides (left and right). Another long and wider projection is visible under the temporal region, which is the articular tubercle of the temporal bone. Above this structure, the external auditory meatus is situated, which is relatively small and oval, with the main axis vertical. The left side only conserved the basal part of this structure whilst the right is more complete, showing the start of the zygomatic process of the temporal bone. However, the connection with the zygomatic bone as well as its upper part is missing. The orbital part of the zygomatic bone is relatively large and extends lateral‐caudally from the maxilla at the level of M^2^. The zygomatic arch is larger than in *S. celebensis* and *S. scrofa vitattus* but thinner and antero‐caudally shorter than in *S. verrucosus*, *S. barbatus*, *S. xiaozhu*, *S. lydekkeri* and *Potamochoerus*.

Both supraorbital foramens are noticeable as well as the longitudinal depressions that correspond to the supraorbital sulcus. The surface of the protuberance defined by these depressions is rough. The snout is long (145 mm), and the lateral borders are situated almost parallel. The dorsal surface of the nasal bone is relatively smooth as in extant *Sus* whilst in *Potamochoerus* it has a rough texture. The infraorbital foramen has a diameter of 7 mm.

#### 3.2.2 Ventral Face

The most remarkable feature in ventral view is the foramen magnum, situated in a more vertical position in comparison with all other extant *Sus* species analysed here and the fossil species *S. lydekkeri*. Consequently, the foramen magnum is completely visible in ventral view. It has a rhombus shape. The left occipital condyle is small and extends down straight (the right is missing in this specimen). The tympanic bulla is pronounced and well‐preserved. The bullae are shorter than in *S. celebensis* but are compressed laterally in both species as well as in *Potamochoerus* spp. The bullae have a straight profile and are slightly inclined antero‐medially towards the muzzle. The jugular process seems not to deviate much laterally from the perpendicular plane, judging from the position of its basal part.

The orbital part of the zygomatic bone extends caudo‐laterally, forming a concavity with a diameter of circa 35 mm. The palatine bone is greatly compressed laterally and shows two greater palatine foramina, one on the right and the other on the left side.

#### 3.2.3 Lateral Face

The temporal bone is almost perpendicular to the transversal axis of the body, and its articular surface (temporal condyle) is situated almost horizontally. From a caudal view, the temporal fossa is convex in its lower region and becomes concave in the upper part. The depression of the temporal fossa is considerably shallower than in *S. verrucosus*, *S. barbatus* and *S. celebensis*. The bone surface of the temporal region is smooth as in the other *Sus* species, unlike in *Potamochoerus* spp., where it is considerably more robust. The tympanic bulla is situated before the maxillary tuberosity. There are two lacrimal foramina at the bottom of the fossa for the lacrimal sac. The supraorbital margin is not connected to the zygomatic process of the frontal bone.

The depression of the maxillary bone is deeper than in *S. celebensis* and *S. lydekkeri* but shallower than in *S. verrucosus*, *S. barbatus*, *S. scrofa vitattus*, and *Potamochoerus* spp. The diastema between the canine and I^3^ is 5 mm. There is hardly any space between the canine and the P^1^.

### 3.3 Upper Dentition

#### 3.3.1 Incisors

I^1^ has a flat labial surface, and slightly curved caudally and downwards. The I^2^ and I^3^ are smaller than I^1^, spatula‐shaped, and have three cusps: the paracone (central), preconule (mesial/anterior), and postconule (posterior/distal). I^2^ is more complex than I^3^ and *S. strozii* type, as figured by van der Made (1996), showing a lingual cingulum, a small accessory cusp on the lingual side. Also, I^2^ has a prestyle in its mesial end and distally a postcrista.

#### 3.3.2 Canine

It is small, spatulated as I^2^ and I^3,^ and bears three cups.

#### 3.3.3 Premolars

The premolars are similar in morphology to *S. barbatus* and *S. celebensis*. P^1^ is small, with three cusps forming a triangle‐shaped cutting edge. P^2^ is small but longer than the P^1^. It has one central cusp (protoconule) that is corrugated on its surface. The paracone and metacone are not distinctly developed. The anterior and posterior occlusal surfaces are oblique and go downwards concerning the high protoconule. Also, it shows small stylids developing in its mesial and buccal faces. P^3^ is larger than P^2^, and is formed by three cusps. The crown shows a pentagonal shape, with the dorsal angle formed by the high protoconule. The paracone and metacone have a similar height, and both are worn and steep, forming a sharp cutting edge with the protoconid. The stylids are more developed in its buccal face than in the lingual. P^4^ is square and shows a strong molarisation trend, but less pronounced than in *S. xiaozhu*. The occlusal surfaces resemble that of the extant *Sus* species following van der Made (1996).

#### 3.3.4 Molars

The molars are not very worn, indicating a young adult specimen. They are robust, bunodont, and brachydont as in extant *Sus*. They have a total of eight accessory cusps: two in the mesial face, and three in their labial and lingual surfaces, respectively, as in *S. barbatus* and *S. celebensis*. M^1^ has a rectangular shape and bears four main cups (protocone, metacone, hypocone, and entocone), as well as the pentaconule. Its occlusal outline forms a clover shape in each quadrant. The hypopreconule is barely visible in the median valley. M^2^ is longer than M^1^ and also rectangular. Apart from the main cusps, the central hypopreconule is completely visible, as well as the anterior protopreconule. The hypoectonule is well developed in M^2^, situated on the labial side, and consists of two small styles. M^2^ has another accessory cusp in the lingual face, defining a small groove between the distal entoconule and mesial metaconule. M^3^ is longer than M^1^ and M^2^. The pentaconules of the talonid of M^3^ are not completely developed, resulting in a triangular shape. The pentapreconule is clearly visible as well as the labial pentaectonule. The hypoconule (labial) and entoconule (lingual) are both clearly visible. It shows the hypoectonule at the labial‐mesial part of the hypoconule. The hypopreconule is flat and situated in the median valley. The protoendoconule is connected with the metaconule. Also, the metaconule shows a small style in its mesio‐lingual end as well as the protoconule.

- RGM. DUB.1860. *Sus brachygnathus*; referred material; male skull. Figure 2.

The skull belongs to an adult male. The skull is well preserved but less complete than RGM. DUB.1862. The back part is missing from the level of the parietal bone. Only the upper part of the left zygomatic arch is conserved. The dental formula is the same as in RGM. DUB.1862. Of the incisors, only both I^1^ are preserved. The skull is similar to RGM. DUB.1862 specimen, with some differences mainly due to sexual dimorphism.

The zygomatic arch is considerably more inflated than in the female specimen RGM. DUB.1860. Additionally, the infraorbital fossa forms a deep and elongated depression, clearly delimited by a relief that extends up and curves slightly in its caudal part. The depression of the canine flanges is shallower than in *S. verrucosus*, antero‐caudally longer and more inflated than in *S. barbatus*, and horizontally shorter than in *S. celebensis*. A foramen (diameter = 10 mm) is present on the dorsal surface of the nasal bone. The diastema between the canine and I^3^ is 10 mm, which is larger than in RGM. DUB.1862. The canine and P^1^ are closely set.

### 3.4 Upper Dentition

#### 3.4.1 Incisors

I^2^ and I^3^ are missing. I^1^ as in RGM. DUB.1862.

#### 3.4.2 Canine

The canines are not preserved, but judging from the position and orientation of its alveolus, the upper canine grows laterally and curves backwards.

#### 3.4.3 Premolars

P^1^ is missing. P^2^, P^3^ and P^4^ are as the specimen RGM. DUB.1862.

#### 3.4.4 Molars

Same as in RGM. DUB.1862. M^3^ is broader, and the talonid is well distinguished in comparison with RGM. DUB.1862.

### 3.5 Mandible

- RGM. DUB.1854 *Sus brachygnathus;* paralectotype; hemimandible dex i3‐m3. Figure 3.
- RGM. DUB.1848 *Sus brachygnathus*; paralectotype; hemimandible sin c‐m3. Figure 4.
- RGM. DUB.1847 *Sus brachygnathus*; referred material; complete mandible. Figure 5.

Specimens RGM. DUB.1854 and RGM. DUB.1848 are the right and left hemi‐mandible, respectively, of a male adult, are well‐preserved and not deformed. Although there are many isolated mandibular fragments of *S. brachygnathus* in the collection, only these two specimens preserve the mandibular ramus. Specimen RUG. DUB.1847 conserve the mandibular symphysis and the incisive part. In dorsal view, the incisive part is concave on both sides up to the canine alveolus, where the maximum expansion of the incisive part occurs. The mandibular symphysis is shorter and horizontally more inclined than in *S. barbatus, S. strozii, S. xiaozhu*, and *S. lydekkeri*. It narrows in the anterior most part, Also, it extends posteriorly to the level of the P_2_. From its buccal face, it descends gradually towards the canine alveolus, from where it descends more abruptly to the alveolar margin, where the symphysis ends. From a caudal view, a V‐shaped depression along the mandibular symphysis can be observed whilst in *S. barbatus*, *S. celebensis*, and *S. strozii* this concavity has a *U*‐shape.

Specimen RGM. DUB.1854 is less well preserved than RGM. DUB.1848, due to the numerous root marks at the bone surface in the lateral face. Also, it presents a relatively high degree of corrosion at its medial face, probably owing to post‐burial chemical processes. For this reason, this specimen is not further considered in the description below.

Hemimandible RGM. DUB.1854 is 250 mm long and has a short and proportionally thick body. The mandibular angle and the condyle are entirely conserved, but the coronoid process, the mandibular notch, and a small fragment of the masseteric fossa are missing, as well as the anterior part of the symphysis.

#### 3.5.1 Mandibular Ramus

The mandibular ramus is lingually concave and labially convex, thin in its central body and increasingly thicker towards the mandibular condyle. The mandibular head is broader rostrally, thinner, and wider antero‐caudally than in *S. verrucosus*. From a dorsal view, the head is oval with the main axis oriented rostro‐caudally in both directions. The inner part has a robust and concave surface: the pterygoid fovea, where the lateral pterygoid muscle inserts.

The lateral face of the ramus is smoother than the medial face and shows a small rugosity for the insertion of the masseteric muscle. The depression of the masseteric fossa is shallower than in *S. verrucosus*, *S. barbatus, S. lydekkeri*, and *Potamochoerus*. The mandibular angle is thick with a rough surface. The insertion area for the medial pterygoid muscle is sharply defined with marked and long rugosities in the medial border of the angle. Also, the mandibular foramen is large and visible on the internal surface of the ramus and has a diameter of 6 mm.

#### 3.5.2 Mandibular Body

The mandibular body is smooth and thinner in its lateral face than in *S. verrucosus*, *S. barbatus, S. strozii*, and *Potamochoerus*, but less inflated, and antero‐caudally longer than in *S. celebensis*. The body has a lightly concave ventral border. A mental foramen is present in the anterior part of the buccal face.

In medial view, a deep depression is present in the ramus, which starts under the M_2_ and has its maximum depth under the M_3_. This concavity is traversed by a bone ridge: the mylohyoid line has a rough texture due to the insertion of the mylohyoid muscle and is deeper than in *S. barbatus* and shallower than in *S. strozii*. Above the mylohyoid line, the surface is smooth.

In the buccal face, a longitudinal prominence is present, with a rough texture and almost subparallel to the tooth row, with its maximum extension in the central part of the lateral face, under the M_1_‐M_2_. This swelling is caused by the protrusion of the canine root inside the ramus.

In occlusal view, the molar part is flattened and elongates rostro‐caudally, becoming considerably convex at the level of M_1_ on its medial face. In the lingual face of the alveolar border, the roots of the premolars and molars are visible.

### 3.6 Lower Dentition

Hemimandible RGM. DUB.1854 retains all the molars and premolars and the canine whilst no incisors (I_1_‐I_3_) are preserved. The root of the canine is exposed. The diastema between P_1_‐P_2_ is concave and considerably longer in comparison with the two other specimens, with a total length of 16.0 mm (7.0 mm in RGM. DUB.1848, 12.0 mm in RGM. DUB.1847). In occlusal view, the teeth from the canine to M_1_ are aligned along an axis inclined caudo‐medially with respect to the long axis of the body. The main longitudinal ridges of the premolars are in line. Dental formula: I 3/3; C 1/1, PM 4/4; M 3/3.

#### 3.6.1 Incisors

Incisors are high‐crowned and are shaped like a rectangular spatula. The occlusal surface is badly preserved due to the strong dental wear and corrosion. I_3_ is missing, exposing the alveolus. The incisors are as in *S. celebensis*, shorter than in *S. verrucosus*, thinner than in *Potamochoerus* spp., but wider than in *S. verrucosus*, *S. barbatus, S. lydekkeri, and B. babyrussa*.

#### 3.6.2 Canine

The canine curves upwards and outwards. Based on the cross‐section of the crown, they can be defined as verrucosic type as in *S. verrucosus, S. barbatus*, *S. celebensis, S. strozii*, and *S. lydekkeri*. It is narrowest at the end of the occlusal face (5 mm diameter) whilst it is broader in its root (12 mm diameter).

#### 3.6.3 Premolars

P_1_ is small with a single cusp, and shorter than that in RGM. DUB.1862. The occlusal border is corrugated, and the buccal and lingual surfaces are slightly convex. The wear surface is oriented obliquely backwards. P_2_ and P_3_ are similar to the P^2^ and P^3^ of RGM. DUB.1862in their lateral face. P_4_ is larger than P_3_ and is more molarised. It has four main cusps and a hypocone that are aligned, forming the main cutting edge. The protocone is the highest cusp in labial view. The stylids at the buccal face are pronounced, especially the anterior one. At the lingual face, the stylids are weakly developed.

#### 3.6.4 Molars

The molars are as in RGM. DUB.1862. M_1_ has exposed dentine due to tooth wear, and the pentaconid is not clearly visible. The pentaconid (distal cusp) of M_2_ is marginally differentiated in this specimen due to wear whilst in RGM. DUB.1848 it is well developed. In M_2_ the hypoectonulid and the accessory cusp at the lingual side (also present in M^2^ of RGM. DUB.1862) are not well discernible. M_3_ is rectangular and slightly curved at its distal end, with a well‐distinguished talonid. The talonid has four cusps as in *S. verrucosus*, *S. barbatus, S. peii*, and *S. lydekkeri*, and is stronger developed than in *S. celebensis*, *S. xiaozhu* and *Potamochoerus* spp. The talonid of *S. brachygnathus* consists of one small terminal cusp (heptaconid) and two taller pairs of cusps: the pentaconid (labial) and the hexaconid (lingual). The terminal cusp of the talonid is divided by a shallow groove.

### 3.7 Sus macrognathus

#### 3.7.1 Systematic Paleontology

Order: Artiodactyla Owen 1848.

Family: Suidae Gray 1821.

Subfamily Suinae Gray 1821.

Genus Sus Linnaeus 1758

Species *Sus macrognathus*; Dubois 1908

Figure 6A–M.

**Figure 6:**
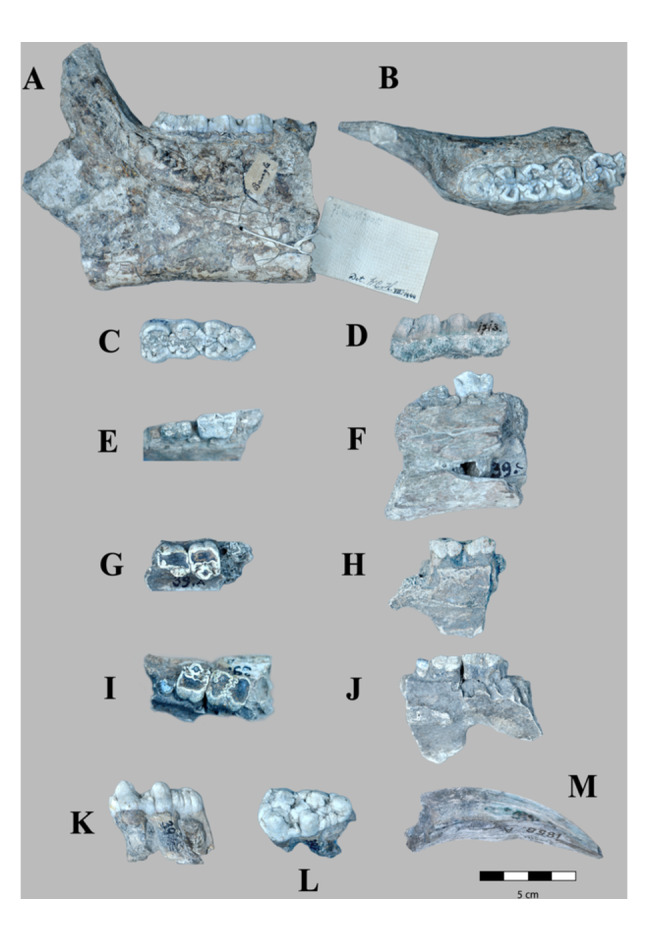
*Sus macrognathus* material from Kedung Brubus, Java (Indonesia; Middle Pleistocene). A, B) left hemimandible with third molar (RGM. DUB.7005a) in lateral and dorsal views. C, D) Lower left M_3_ (RGM. DUB.1713; lectotype) in occlusal and lateral views; E, F) right hemimandible with third and fourth premolar (RGM. DUB. 39c) in occlusal and lateral views G, H) left maxilla with third and fourth premolar (RGM. DUB.39a) in occlusal and lateral views; I, J) right maxillary with fourth premolar and first molar from a male individual (RGM. DUB.39b) in occlusal and labial views; K, L) isolated upper right third molar (RGM. DUB.39e) in lateral and occlusal views; M) isolated lower right canine of a male individual (RGM. DUB.1858a).

Synonyms:

*Sus verrucosus* Dubois 1891

*Sus macrognathus* Dubois 1908.

*Sus coerti* von Koenigswald 1934

*Sus* Badoux 1959.

*Sus macrognathus* Hardjasasmita 1987.

#### 3.7.2 Known Distribution

Type locality: Kedung Brubus, Java. Horizon: Middle Pleistocene ( ~ 0.8–0.7 mya). Occurrence: Bangle, Tritik, Kedung Brubus and Toeanan.

#### 3.7.3 Material Examined

Lectotype: RGM. DUB.1713: M_3_ inf sin. D. Established by Hardjasasmita (1987).

RGM. DUB [7005a (mandible sin. M_3_); 1858a (canine inferior dex; no type); 39b (maxilla dex P^4^‐M^1^,); 39c (mandibula dext P_3‐_P_4_,), 39e (M^3^ sup dex,); 39a (maxilla sin P^3^‐P^4^, no type)]

Specimens RGB. DUB.7005a, 39b, 39e and 39c are referred to as syntypes 1‐4 in Hardjasasmita (1987), but are considered paralectotypes, following the designation of the lectotype.

#### 3.7.4 Revised Diagnosis

Large‐sized species of pig. Teeth are more elongated than in *S. brachygnathus*. The lower male canine is of the verrucosic type. The dental formula is as in *S. brachygnathus*. Premolars are large and well‐developed. The lower premolars have a cutting surface, but the upper premolars are square and molarised. The upper molars are morphologically as in *S. brachygnathus* and extant *Sus*, but larger and wider. Lower molars are significantly longer than in other extant *Suinae*, especially M_3,_ which is distally expanded. M_3_ is larger than in *S. verrucosus* and has three pair of cusps. The talonid is similar to that in *S. verrucosus*, but with an extra cusp in the median valley (heptapreconulid). The enamel of M_2_ and M_3_ is thinner than in *S. brachygnathus*, extant *Sus* and *Potamochoerus* spp., and their occlusal morphology is comparable with extant *Phacochoerus*, with a tendency to develop transversal ridges (‘lophed’ or ridged‐type molars)

### 3.8 Anatomical descriptions

#### 3.8.1 Mandible

- RGM. DUB.7005a. *Sus macrognathus*; paralectotype; hemimandible sin with M_3_. Figure 6A,B.

The specimen consists of the posterior part of the mandibular ramus with the M_3_ preserved and the posterior part of the M_2_. Overall, the mandibular body is not well preserved due to damage to the bone surface caused by the action of roots, weathering (stage 0–1, following (Behrensmeyer 1978), and discoloration due to chemical agents (probably precipitated MnO_2_ and limonite).

The mandibular ramus is robust, thick, and slightly concave laterally and shows a projection at the level of M_2,_ likely due to the root of the canine. The coronoid process, the head of the mandible, and the mandibular angle are missing. The mylohyoid line is strong and well‐defined. The depression between the posterior end of M_3_ and the upgoing part of the mandibular ramus is deep and relatively large. In dorsal view, the molars are slightly bulge out laterally and are aligned along the same axis.

#### 3.8.2 Lower Dentition

#### 3.8.3 Canine

- RGM. DUB.1858a. *Sus macrognathus*; referred material; Canine inferior dex. Figure 6M.

The canine is of the verrucosic type as in *S. verrucosus, S. barbatus*, *S. celebensis, S. strozii, S. lydekkeri*, and considerably wider and larger than in *S. brachygnathus*.

#### 3.8.4 Premolars

- RGM. DUB.39.c *Sus macrognathus*; paralectotype; mandibula dex P_3_‐P_4_. Figure 6E,F.

P_1_ and P_2_ are not preserved. P_3_ is considerably damaged, and the occlusal surfaces cannot be appreciated in detail. P_4_ is well preserved and shows a strong molarisation trend as in *S. verrucosus*. P_4_ has two main cusps. The protoconid in the central‐mesial part is higher than the distal metaconid, forming a cutting edge (triangular‐shaped). It also has two stylids on the mesial and distal sides of both faces (buccal and lingual), more pronounced than in *S. brachygnathus*.

#### 3.8.5 Molars

- RGM. DUB.1713 *Sus macrognathus*; lectotype; M3 inf sin. Figure 6C,D.
- RGM. DUB.7005.a *Sus macrognathus*; paralectotype; mandible sin. M2‐M3. Figure 6A,B.

The molars are robust, bunodont, and brachydont as in *Sus* and *Potamochoerus*, with a tendency to develop transversal ridges (‘lophed’ type) as in *S. strozii* and *Phacochoerus aethiopicus*. Based on how the individual cusps are linked to form these ridges, this morphology has been described as a columnar type (Janis 2008). As in *S. verrucosus*, the molars have fewer accessory cusps than in *S. brachygnathus*, *S. barbatus*, and *S. celebensis*.

M_1_ is not preserved. M_2_ is partially preserved in RGM. DUB.7005a. M_3_ is considerably longer than in all the other extant and fossil *Sus* analysed here. It bears three pairs of cusps and a talonid at the posterior end. The enamel is considerably thick, showing folds (lowered areas) that develop from each cusp. Transversal ridges separate the cups from each other, showing as well‐marked invaginations There are three well‐developed cusps in the median valley: the protoendoconulid, situated behind the most anterior pair of cusps. The second cusp (the hypopreconulid) is in a central position at the anterior part of the talonid and the pentapreconulid posteriorly. The talonid is more developed than in *S. brachygnathus*, having a well‐defined heptapreconulid in the median valley. It has a triangular shape and is formed by the third row of cusps and a large median cusp (pentapreconulid) that is divided transversally into two separated small cusps. There is a small stylid, barely visible, situated at the posterior end of the lingual face. The distal end of the talonid consists of two smaller cusps.

### 3.9 Upper Dentition

- RGM. DUB.39.b *Sus macrognathus*; paralectotype; maxilla dex P4‐M1. Male individual. Figure 6I,J.
- RGM. DUB.39.e Sus ma*crognathus*; paralectotype; M^3^ sup dex. Figure 6K,L.

#### 3.9.1 Premolars

- RGM. DUB.39a. *Sus macrognathus*; referred material; maxilla sin P^3^‐P^4^. Figure 6G,H.

Both premolars (P^3^ and P^4^) are lophodont and are worn. P^3^ has a cusp in its lingual face, formed by one main median cusp with two small styles at both ends. This accessory cusp forms a protuberance and is clearly distinguished from the main cusp. P^4^ is square and has a higher molarisation degree than P_4._ It has a well‐developed central‐lingual cusp. Lingually, from the main cusp of the P^4^ is an antero‐posteriorly orientated fold.

#### 3.9.2 Molars

M^2^ is not preserved. M^1^ is larger than P^4^ but has the same width. In occlusal view, M1 is divided into two parts separated by a wavy, well‐defined enamel ridge. It has a small, round accessory cusp at the lingual‐mesial border.

- RGM. DUB.39.e *Sus macrognathus*; paralectotype; M^3^ dex. Figure 6K,L.

This M^3^ is well preserved and considerably less worn than in M^3^ is shorter than M_3_ and less complex. Also, the transversal ridges are not present, and morphologically, it is a typical bunodont tooth. In occlusal view, it has an isosceles triangle shape. It consists of four main cusps situated laterally and a talonid. The mesial cingulum is well developed. Apart of the four main cusps, there are also two median accessory cusps: the anterior hypopreconulid, situated after the first pair of cusps, and the posterior pentapreconulid. Along the labial border, there are six accessory cusps: one anteriorly, in front of the hypopreconulid, and another small five posteriorly, situated after the second pair of cusps, forming the outer rim of the talonid. On the lingual side, there are only two small styles.

## 4 Discussion

Few phylogenetic studies on extinct and extant *Sus* species are based on morphological features (e.g., Hardjasasmita 1987; Cherin et al. 2018). However, a solid consensus on craniodental morphological traits that are phylogenetically relevant within *Sus* is lacking. Some authors (Cucchi et al. 2009) suggest that the high plasticity of the M_3,_ especially in its posterior part (talonid) can be a valuable approach to address taxonomy among closely related taxa. They analysed M_3_ shape variability within ISEA extant wild pigs and found that all *Sus* species and subspecies can indeed be differentiated based on the shape of the M_3_. They recognise two main groups: 1) the warty wild pigs (*S. celebensis* and *S. philippensis*) and 2) the Sundaic wild pigs that in their turn can be subdivided into two subgroups: 1) *S. barbatus* and 2) *S. verrucosus/S. scrofa vittatus*. These groups mostly differ in the configuration of the talonid. The first group is characterised by a nonsymmetric M_3_ and a simplified talonid lacking a heptaconid. Following this definition, both *S. brachygnathus* and *S. macrognathus* would be within the Sundaic wild pig group due to the presence of a heptaconid.

The first phylogenetic analysis of both extant and extinct Suinae species based on craniodental morphological traits (52 in total) was carried out by Cherin et al. (2018). They placed *S. brachygnathus* as a sister group to *S. celebensis*, forming a clade (unlike the placement following Cucchi et al. 2009), but the relationship between them and the ISEA pigs, including *S. verrucosus* and *S. barbatus*, was not resolved. The presence of this polytomy reflects the difficulty of finding apomorphic and plesiomorphic craniomandibular traits to differentiate these lineages, which can be explained by their rapid radiation in the region and hybridisation processes favoured by sea‐level oscillations during the Pleistocene. Therefore, conclusions concerning the phylogenetic relationships of these taxa are rather speculative, as both morphological characters and molecular data may be altered due to these admixture events between ISEA pigs. In comparison with the extant *Sus* species, *S. brachygnathus* undoubtedly has a skull morphotype fairly similar to *S. celebensis*. The affinity of both species was already noticed earlier (von Koenigswald 1933; Cherin et al. 2018). However, from the anatomical description provided here, it appears that S*. brachygnathus* has distinctive skull features within Suinae, which are: 1) a low position of the foramen magnum, 2) a straight dorso‐ventral profile of the skull, and consequently, 3) low head/parietal‐occipital region with a shallow temporal fossa. These traits are not necessarily phylogenetically informative but likely have ecological and morpho‐functional implications. Further study is needed to resolve this, which is outside the scope of this contribution.

Regarding tooth morphology, both Javanese fossil species share the typical *Sus* traits, being characterized by well‐developed premolars, bunodont and brachydont molars with many accessory cusps and a well‐defined talonid. These morphological features are present in extant omnivorous *Sus*, adapted to process a wide range of food sources (Souron 2017). *Sus brachygnathus* shows the highest affinity with *S. barbatus*, not only in the configuration of the molar main cusps but also in the distribution and number of accessory cusps. The similarity of these species was already pointed by Badoux (1959), who argued that they were synonymous, even though both species differ considerably in skull morphology and size. In contrast, *S. macrognathus* shows a high morphological similarity with *S. verrucosus*, but they differ significantly in the third lower molar. *Sus macrognathus* has a peculiar M_3_, which is distally expanded and bearing distinct grooves (‘lophs’), similar to those found in the African suid *Phacochoerus aethiopicus*, though not as pronounced as in that lineage. The tendency toward more transverse enamel folds, anteroposterior elongation of the M_3_ and the increase in number of cusps in *S. macrognathus* is likely an adaptative response to an environmental pressure and might be indicative of a more abrasive diet (Jernvall et al. 1996; Janis 2008) compared to other *Sus* species. Similar morphological traits are found in extant African species with predominantly herbivorous feeding habits (Souron 2017). Additionally, the large body size of *S. macrognathus* is in line with a trend toward incorporating more abrasive food items within its omnivorous diet. However, more research is required to support these ecological inferences.

**Figure 7:**
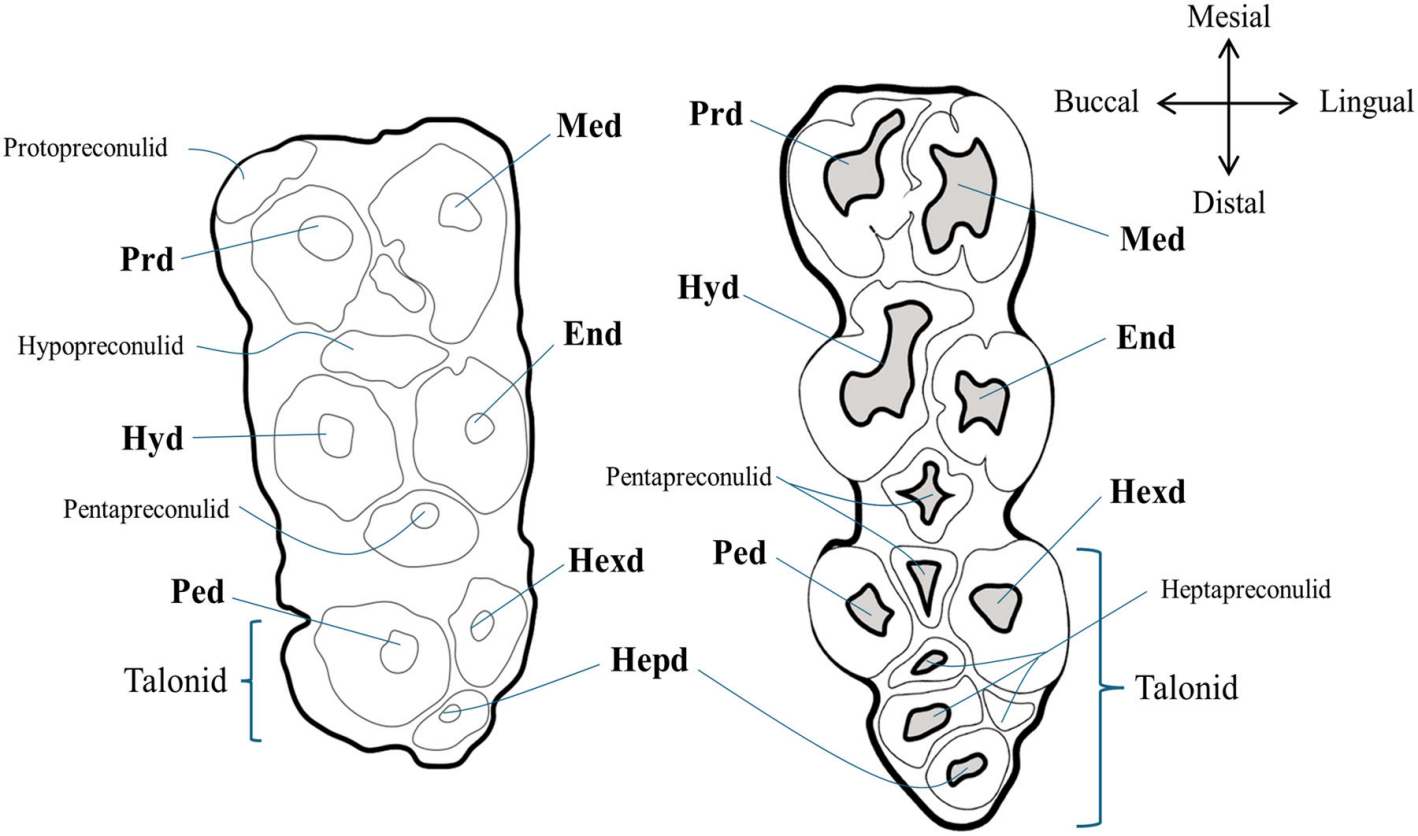
Third lower molar of *S. brachygnathus* (left) and *S. macrognathus* (right) with the main cups and their morphological terminology. End, entoconid; Hepd, heptaconid; Hexd, hexaconid; Hyd, hypoconid; Med, metaconid; Pad, paraconid; Ped, pentaconid; Prd, protoconid.

Regarding dental traits, the two extinct Javanese pig species differ mostly in size and the morphology of the lower molars, especially in the M_3,_ as was mentioned above. Apart from the overall morphology, the talonid of the M_3_ in S*. brachygnathus* consists of two main cusps with one heptaconid terminally whilst *S. macrognathus* incorporates the third pair of main cusps into the talonid and has a deeper developed talonid valley, showing an additional, well‐defined accessory cusp (heptapreconulid), which is laterally and medially expanded (see Figure 7). These marked differences in M_3_ morphology between these species is in line with Cucchi et al. (2009), who propose the use of M_3_ as a phenotypic marker because of its high plasticity in suids.

At present, the phylogenetic relation between the two extinct Javanese pig species cannot be fully resolved. Two possible scenarios can be hypothesised: 1) *S. macrognathus* evolved from *S. brachygnathus* in situ following a trend of (pen)insular gigantism, 2) the two species represent independent dispersals to Java at successive time periods. Hardjasasmita (1987) considered both species contemporaneous, and discussed these two scenarios accordingly, but later research (van den Bergh et al. 2001; van der Geer et al. 2021) has shown that *S. brachygnathus* is restricted to the geologically older Trinil H.K. fauna and *S. macrognathus* occurs in geologically younger layers, belonging to the Kedung Brubus and Ngandong biozones.

The first scenario would explain the increment in body size between the two species in a relatively short period. Although insular pigs are expected to follow a trend of decreasing body mass (Lomolino et al. 2013), as is the case with the Sulawesi warty pig (*S. celebensis*) and the Flores warty pig (*S. celebensis floresianus*), a large‐sized pig is known from the Early Pleistocene of Sulawesi (*Celebochoerus heekereni*; Hooijer 1948; van der Geer et al. 2021). A secondary body size decrease has been observed in the Early Pleistocene pig from Sardinia *Sus sondaari* van der Made 1999 (van der Made 1988) with respect to its putative ancestor *S. arvernensis* (van der Made et al. 2006; Iannucci et al. 2021; Iannucci 2024), representing the only clear case of insular dwarfism among fossil suid species.

The environmental conditions in Java changed numerous times during the Pleistocene (van den Bergh et al. 1996, 2001; van der Geer et al. 2021) due to glacial cycles, hence *S. brachygnathus* could have evolved into *S. macrognathus* relatively fast due to environmental pressures and favoured by the isolation, representing an adaptative evolution by chronospecies. This could explain the morphological differences between the two species, which seems to give an ecological signal rather than being phylogenetically informative. For instance, in European *Sus scrofa*, ecomorphological variations such as size changes related to glacial–interglacial cycles from the late Middle Pleistocene to the Early Holocene have been documented (Iannucci et al. 2020). Another illustrative example is *S. sondaari*, which displays notable morphological changes from its mainland ancestor, such as the development of a more hypsodont dentition as an adaptation to an herbivorous diet (van der Made 1988, 1999; Palombo et al. 2012). However, such pronounced morphological changes in *S. sondaari*, favoured by the selective pressures of an isolated insular environment, are much less evident in ISEA pigs. This contrast may be explained by the degree of isolation. The recurrent connectivity of ISEA islands during the Pleistocene glaciations facilitated migrations and hybridisation events, leading to relatively lower ecomorphological divergence among ISEA pigs.

The second scenario would involve two independent dispersals and allopatric evolutionary processes. Two populations of *Sus*, probably closely related to *S. scrofa* considering the phylogenetic evidence based on DNA analysis that situates *Sus scrofa* near/at the root of the S*us* node (Gongora et al. 2011), migrated from the Asian mainland to Java at different glacial stages, followed by subsequent isolation during an interglacial stage, resulting into *S. brachygnathus* and *S. macrognathus*, respectively. In any case, both species are endemic to Java, because thus far there is no fossil evidence for either of the two species anywhere outside Java.

To properly test the two scenarios, however, more complete remains of *S. macrognathus* as well as a revision of phylogenetically valid traits in *Sus* are necessary. The anatomical descriptions and comparisons presented here provide a first step for future systematic research, regarding the phylogenetic relationship between these two species, ISEA pigs and Asian mainland species, including modern *S. scrofa* and ancestral forms.

## 5 Conclusion

The two extinct Javanese suid species, *Sus brachygnathus* and *S. macrognathus*, differ morphologically from extant suid species. Although *S. brachygnathus* shows a skull morphotype similar to *S. celebensis*, it exhibits unique features within the Suinae considered in this study. *Sus macrognathus* differs mainly in its M_3_ from the extant species. The two fossil species differ mostly in size and in the morphology of the lower molars, with marked differences in the M_3_. This distinction in M_3_ configuration is in line with previous research that proposes it as a phenotypic marker to address taxonomy among closely related taxa. Besides that, the differences in tooth morphology seem to give an ecological signal. *S. macrognathus* shows morphological features that indicate a more abrasive diet compared to the other *Sus*. Currently, the phylogenetic relationship between the two extinct pig species cannot be fully resolved. Two evolutionary scenarios are proposed: 1) the evolution of *S. macrognathus* from *S. brachygnathus* through an insular gigantism trend, or 2) the two species represent independent dispersals from the Asian mainland to Java at successive periods. Our results add to the paucity of studies on the morphological disparity of Suinae based on comparative anatomy traits. It constitutes a firm morphological basis for future phylogenetic and taxonomic studies in both extant and extinct *Sus* based on craniodental morphology.

## Author Contributions

**Rachel V. Pacheco‐Scarpitta:** investigation, writing – original draft, writing – review and editing.

### Peer Review

The peer review history for this article is available at https://www.webofscience.com/api/gateway/wos/peer-review/10.1002/jmor.70057.

## Data Availability

The data that support the findings of this study are available on request from the corresponding author. The data are not publicly available due to privacy or ethical restrictions.
